# Mitigating Milk-Associated Bacteria through Inducing Zinc Ions Antibiofilm Activity

**DOI:** 10.3390/foods9081094

**Published:** 2020-08-11

**Authors:** Carmel Hutchings, Satish Kumar Rajasekharan, Ram Reifen, Moshe Shemesh

**Affiliations:** 1 Department of Food Science, Institute for Postharvest Technology and Food Sciences, Agricultural Research Organization (ARO), Volcani Center, Rishon LeZion 7505101, Israel; carmel100@gmail.com (C.H.); generic.sat@gmail.com (S.K.R.); 2 The Robert H. Smith Faculty of Agriculture, Food and Environment, Institute of Biochemistry, Food Science and Nutrition, The Hebrew University of Jerusalem, Rehovot 7610001, Israel; ram.reifen@mail.huji.ac.il

**Keywords:** dairy industry, biofilm, *Bacillus*, minerals, zinc, alternative antibacterial treatments

## Abstract

Dairy products are a sector heavily impacted by food loss, often due to bacterial contaminations. A major source of contamination is associated with the formation of biofilms by bacterial species adopted to proliferate in milk production environment and onto the surfaces of milk processing equipment. Bacterial cells within the biofilm are characterized by increased resistance to unfavorable environmental conditions and antimicrobial agents. Members of the *Bacillus* genus are the most commonly found spoilage microorganisms in the dairy environment. It appears that physiological behavior of these species is somehow depended on the availability of bivalent cations in the environment. One of the important cations that may affect the bacterial physiology as well as survivability are Zn^2+^ ions. Thus, the aim of this study was to examine the antimicrobial effect of Zn^2+^ ions, intending to elucidate the potential of a zinc-based antibacterial treatment suitable for the dairy industry. The antimicrobial effect of different doses of ZnCl_2_ was assessed microscopically. In addition, expression of biofilm related genes was evaluated using RT-PCR. Analysis of survival rates following heat treatment was conducted in order to exemplify a possible applicative use of Zn^2+^ ions. Addition of zinc efficiently inhibited biofilm formation by *B. subtilis* and further disrupted the biofilm bundles. Expression of matrix related genes was found to be notably downregulated. Microscopic evaluation showed that cell elongation was withheld when cells were grown in the presence of zinc. Finally, *B. cereus* and *B. subtilis* cells were more susceptible to heat treatment after being exposed to Zn^2+^ ions. It is believed that an anti-biofilm activity, expressed in downregulation of genes involved in construction of the extracellular matrix, would account for the higher sensitivity of bacteria during heat pasteurization. Consequently, we suggest that Zn^2+^ ions can be of used as an effective antimicrobial treatment in various applications in the dairy industry, targeting both biofilms and vegetative bacterial cells.

## 1. Introduction

Dairy products constitute one of the leading sectors impacted by food loss [1,2]. Bacterial contamination can adversely affect the quality, functionality and safety of milk and its derivatives. It appears that one major source of contaminations of dairy products is often associated with the formation of biofilms on the surfaces of milk processing equipment [3]. Biofilms are a multistage process in which bacterial cells adhere to a surface and/or to each other through production of an extracellular matrix, which surround and subsequently protect the bacteria [4,5]. Once the biofilm is established, bacterial cells within are characterized by increased resistance to unfavorable environmental conditions, antimicrobial agents, and cleaning solutions and procedures [6,7]. After the establishment of the surface-attached biofilms, their removal can be extremely challenging. Their extraordinary resistance to antibacterial treatments is attributed to several factors, including inability of the antimicrobial agent to fully penetrate the biofilm through the matrix [8,9,10].

The composition of milk makes it an ideal medium for the growth of microorganisms preferentially in the biofilm mode [11]. Biofilm bacteria can be found on virtually all types of product contact surface; from milk cups in dairy farm to heat exchangers in the processing plant [12]. Moreover, bacteria can also form biofilm bundles during growth within the milk [13]. Bundles can attach to the surface of the dairy equipment or circulate through the milking pipelines, enabling biofilm dispersal throughout the processing equipment [14]. The persistent accumulation of bacteria in the form of biofilms on dairy equipment eventually leads to a shorter shelf life of products and in some cases transmission of diseases [6,15].

*Bacillus* species are Gram-positive, motile, rod-shaped, spore-forming bacteria regularly found in soil. Members of the *Bacillus* genus are among the most common bacteria found in dairy farms and processing plants [10,16]. Furthermore, members of the *Bacillus subtilis* and *Bacillus cereus* groups are the most important spoilage microorganisms in the dairy environment [17]. Both groups produce various extracellular heat-stable enzyme, which contribute to the reduction of shelf life of processed milk and dairy products by degradation of milk components and additives [18]. Since *B. cereus*, along with other members of the *Bacillus* genus, are human pathogens, bacterial presence can also present a health risk once consumed [18,19,20,21].

*Bacillus subtilis* is a non-pathogenic bacterium with a complex regulatory system destined to coordinate expression of genes encoding extracellular matrix in response to changes in environmental conditions [22]. The *B. subtilis* matrix has two main components; one component is exopolysaccharides, synthesized by the products of the *epsA-O* operon, which is essential for the cells bundling [13,23]. The other matrix component is amyloid-like fibers encoded by *tasA* located in the *tapA-sipW-tasA* operon. The same operon also encodes for TapA, a structural protein that presumably attach the amyloid-like fibers of the biofilm to the cell wall. The functions of EPS and TasA within the bundle are different. EPS is required for side to side attachment of cell chains and is necessary for the formation of biofilm bundles. Meanwhile, TasA seems to fine-tune the folding properties of the bundles. In this way, matrix-producing cells organize themselves into multicellular structures [24].

The common method to control biofilm formation in the food industry nowadays involves the use of frequent cleaning procedures in combinations with disinfectants [3]. On its own, cleaning leads to the removal of approximately 90% of the bacteria from the surfaces, but it does not kill them [25]. Bacteria might reattach later to the surface and form a new biofilm, hence integration of disinfectants in the cleaning procedure is indispensable. As previously mentioned, conventional cleaning and disinfection regimes can be ineffective due to failure of fully penetrating the biofilm matrix [9,10]. Therefore, it appears that ideally cleaning should be carried out in a way that damage the exopolysaccharides matrix, so that the disinfectants can gain access to the bacterial cells within [9]. Due to the increased resistance of biofilms to conventional disinfection processes, new and novel control strategies are constantly sought after. Since microorganisms can also develop resistance to substances, and subsequently survive previously effective procedures, routine control regimes may turn ineffective after a certain given time [11]. Therefore, there is a need for development of other methods that prevent and eradicate bacterial biofilms efficiently.

Zinc is an essential mineral crucial for cellular functions and therefore indispensable for the biochemistry of life in all organisms, including bacterial cells. However, this metal ion can also be toxic to bacterial cells when presented in high concentrations [26,27]. Zinc seems to have a wide range of antimicrobial activity; past studies have shown that elevated concentrations of zinc inhibited the growth of several bacterial strains and limited bacterial conjugation [28,29,30]. Moreover, Zinc has been found to inhibit biofilm formation by several Gram-positive and Gram-negative bacteria [30]. The mechanism behind the antibiofilm activity of zinc has yet to be characterized, but it has been suggested that zinc could interact with components of the matrix [31]. Furthermore, zinc may also interfere with other cellular mechanisms such as signaling and gene regulation [32]. Zinc’s wide range of anti-infectious abilities has subsequently led to the use of this mineral in various applications that requires contamination and infection control [33,34]. Our study sought to test whether zinc addition can provide a solution to the microbial contaminations that are frequently emerging in the dairy industry. Thus, the aim of this study was firstly to investigate the effect of Zn^2+^ ions on biofilms formed by *B. subtilis*. Secondly, to investigate the molecular basis of zinc’s effect on *B. subtilis* cells, focusing on genes encoding for the extracellular matrix and its components. Thirdly, to examine the potential of zinc-based antibacterial activity in conjunction with classical heat treatment.

## 2. Materials and Methods

### 2.1. Strains and Growth Conditions

The *Bacillus subtilis* wild strain NCIB3610 [35] and *Bacillus cereus* ATCC 10987 strain, obtained from Michel Gohar’s lab collection (INRA, Jouy-en-Josas, France), were used in this study. For fluorescent microscopy, we used the fluorescently tagged *B. subtilis* strains (YC161 and YC189). The YC161 strain produces GFP constitutively (P*spank*-*gfp*), while YC189 harbors a gene coding to cyan fluorescent protein (CFP) under the control of the *tapA* promoter (P*tapA*-*cfp*) [36,37]. Both strains were obtained from the laboratory collection of Yunrong Chai (Northeastern University, Boston, MA, USA). For routine growth, all strains were propagated in Lysogeny broth (LB) (Difco) comprising (per liter): 10 g of tryptone, 5 g of yeast extract, and 5 g of NaCl or on solid LB medium supplemented with 1.5% agar. Prior to generating starter cultures, bacteria were grown on the agar-solidified plates overnight at 37 °C. A starter culture of each strain was prepared using a single bacterial colony; for starter cultures, bacteria were inoculated from the agar plates into 5 mL of LB broth and incubated at 23 °C with shaking at 150 rpm overnight. For biofilm generation, a starter culture was diluted 1:100 into 10 mL of LB and incubated overnight at 30 °C with shaking at 50 rpm. In order to improve biofilm formation, we used LB medium supplemented with 3% lactose (Difco), since it was recently marked as a potent inducer for formation of biofilm bundles by *B. subtilis* [38]. Use of LB-lactose rather than milk as the growth media was designed in order to achieve a clearer viewing of the bacteria cells microscopically.

A comparison between the antimicrobial activity of the various generally recognized as safe (GRAS) approved zinc compounds did not show a significant difference. Therefore, zinc chloride was chosen as the source for the zinc ions, being highly soluble and stable as a solution. Use of Zn^2+^ ions in all assays was done by diluting a 1M solution of ZnCl_2_ (Sigma-Aldrich, St. Louis, MI, USA) into the growth medium to achieve a desirable final concentration. The effect of Zn^2+^ ions on biofilm was evaluated in two aspects- effect on biofilm formation and effect against already formed biofilm. To investigate the effect of Zn^2+^ ions on biofilm formation, ZnCl_2_ was added to the medium along with the diluted bacteria at concentrations of 0.1, 0.2, 0.3 mM. Those concentrations were previously found in our lab to have no significant effect on bacterial growth (Figure S1), making them suitable to test zinc’s effect on biofilm formation. Un-supplemented samples were used as controls. To investigate the effect of Zn^2+^ ions on disruption of biofilm, ZnCl_2_ was added to the medium at concentrations of either 3 or 5 mM, after 17 h in which bacteria was grown at the previously mentioned conditions. Incubation period of 17 h was set in order to grant cells enough time to develop sufficient number of the biofilm bundles. Un-supplemented samples were used as controls.

### 2.2. Visualizing Biofilm Bundles Using Confocal Laser Scanning Microscopy (CLSM)

For visualization of *B. subtilis* biofilm bundles, we used the fluorescently tagged *B. subtilis* strain (YC161), which produces GFP constitutively [37]. The biofilm bundles were generated as described above. To prepare the samples for microscope observation, 1 mL of each sample was collected and centrifuged at 10,000× *g* for 2 min. The supernatant was discarded, the pellet was washed with sterile distilled water and then suspended in 40 μL of sterile distilled water. Then, 7 μL of each sample were transferred onto glass slides and visualized with a SP8 confocal laser scanning microscope (CLSM) (Leica, Wetzler, Germany) equipped with a HC PL APO 40x/1.1 water immersion objective (Leica, Wetzlar, Germany) and 488 nm laser for GFP excitation. To investigate the effect of Zn^2+^ ions on biofilm formation, a sample was taken from the control and from each concentration after an overnight incubation of 20 h in the presence of zinc. To conceptualize the changes in bundle size caused by exposure to zinc, images were further analyzed for cell density using Image J 1.x software (Bethesda, Maryland, USA). Cell density was calculated as the number of visible cells per area. To investigate the effect of Zn^2+^ ions on dispersion of biofilm, a sample of each concentration was taken after 17 h of incubation, prior to zinc addition, in order to assure biofilm formation was successful. A second sample was taken for microscopic visualization after an additional incubation of 5 h in the presence of zinc.

### 2.3. Examination of Expression of Matrix Gene in B. subtilis Using CLSM and Florescence Microscope

To determine the expression of the extracellular matrix components, essential for biofilm formation and survival, another fluorescently tagged *B. subtilis* strain (YC189) was used. This strain harbors a gene coding to cyan fluorescent protein (CFP) under the control of the *tapA* promoter [35]. Hence, CFP expression in this strain indicates that the *tapA* operon is being activated and fluorescently visible cells are in a biofilm state. CFP expression was observed using 458 nm laser on CLSM or using 436 nm laser using Eclipse Ti2 inverted microscope (Nikon, Shinagawa, Japan). Generation of biofilm bundles and preparation for microscope observation were performed as previously described. Assays for evaluation of the effect on biofilm formation and the effect on disruption of biofilm were also done as described in the previous section. Measurement of fluorescence intensity of cells expressing the *tapA* operon was attained using ImageJ 1.x software (Bethesda, ML USA). Relative expression of *tapA* operon was measured and normalized per cell density.

### 2.4. RNA Extraction and Real-Time Reverse Transcription PCR

In order to further examine the effect of Zn^2+^ ions on expression of biofilm related genes, we chose to use the real time RT-PCR method for genes of the matrix operons *epsA-O* and *tapA-sipW-tasA*. Initially, *B. subtilis* NCIB3610 cells were grown to the late-log phase for 5 h at 37 °C In shaking culture at 150 rpm, in LB medium. Next, cultures were diluted 1:100 into 6 mL LB-lactose medium supplemented with ZnCl_2_ in concentrations of either 0.2 or 0.3 mM, while un-supplemented samples were used as controls. The samples were incubated overnight at 23 °C with shaking at 50 rpm. After the overnight incubation, 3 mL from each sample was collected and centrifuged at 5000× *g* for 10 min. RNA was extracted using the Qiagen RNeasy mini Kit (QIAGEN, Hilden, Germany), according to the manufacturer protocol. The RNA concentration was determined spectrophotometrically using the Nanodrop-2000 instrument (ThermoFisher Scientific, Waltham, MA, USA). cDNA was synthesized from 1 μg RNA in reverse transcription reaction using qScript cDNA Synthesis Kit (Quantabio, Beverly, MA, USA) according to the manufacturer’s instructions. All cDNA samples were stored at −20 °C. RT-PCR reactions (final volume = 20.0 μL) consisted of 2 μL cDNA template, 10 μL fast SYBR green master mix, 1 μL suspension of each primer, and 7 μL RNase free water. Forward and reverse PCR primers (Table S1) were designed using the Primer express software and were synthesized by hay-labs (Rehovot, Israel). DNA was amplified with the Applied Biosystems StepOne™ Real-Time PCR System (Life technologies, Foster, CA, USA) under the following PCR conditions: denaturation 2 min at 95 °C and 40 cycles of 95 °C for 3 s, 60 °C for 30 s, and 95 °C for 15 s. RNA samples without reverse transcriptase were used as negative control, to determine there is no DNA contamination. The expression level of the tested genes was relatively calculated using 16S rRNA and *rpoB* genes as endogenous control (Table S1).

### 2.5. Visualization of Morphological Changes in Bacterial Cells Exposed to Zinc Using Scanning Electron Microscopy (SEM)

For microscopic visualization of *B. subtilis*, cells of strain NCIB3610 were first grown for 5 h in LB medium at 23 °C with shaking at 150 rpm. Next, cultures were diluted 1:100 into 10 mL LB medium and zinc was added at concentrations of either 0.2 or 0.3 mM for an overnight incubation. In order to investigate the effect of higher concentrations of zinc on bacterial cells, zinc was added in concentrations of either 3 or 5 mM for the duration of 4 h, with samples being taken at two points: after 2 h and at the end of the incubation period. At first, samples were prepared for microscopic observation as previously described. After suspension in sterile DW, 3 µL of each sample was placed on polylysine-coated glass discs for 1 h, then washed with DDW in order to remove unattached cells. Cells were then fixated using 4% glutaraldehyde, and once again washed three times with DDW. The glass discs were then treated with a series of dehydration and drying procedures, intending to replace the previously used DDW with alcohol, as a final step before scanning electron microscopy (SEM) visualization. The microphotographs were recorded using scanning electron microscope JEOL model, JSM-IT-100 LV (JEOL, Tokyo, Japan). The images were taken with an accelerating voltage of 5–10 kV, at high vacuum (HV) mode and secondary electron image (SEI). Obtained images were than analyzed using ImageJ software and cells were measured in order to estimate differences in cell size.

### 2.6. Analysis of Survival Rates Following Heat Treatment

Starter cultures of *B. cereus* ATCC 10987 and *B. subtilis* NCIB 3610 were prepared as described above. The cultures were then diluted 1:100 into LB medium and grown for an additional 5 h at 37 °C with 150 rpm. Next, zinc was added to each sample at concentrations of either 3 or 5 mM, with un-supplemented samples representing the controls. Samples were then incubated for 3 h at room temperature of 25 °C. The samples were then heat treated for 30 s at 72 °C in a water bath. The number of surviving cells after heat treatment was quantified by the CFU method, i.e., 100 µL of serial dilutions from each sample were spread-plated on 1.5% LB agar and incubated overnight at 37 °C. Prior plating the samples were subjected to mild sonication procedure: for 20 s—amplitude, 20%; pulse, 10s; pause, 10s—with an Ultrasonic processor (Sonics, VCX 130, Newtown, CT, USA). Total CFU per ml of each sample was calculated from the number of colonies derived after an overnight incubation.

### 2.7. Statistical Analysis

The data obtained were analyzed statistically by means of ANOVA following post hoc t-test using Microsoft Excel 2010, JMP 10, and GraphPad prism 6 software. *p*-values less than 0.05 were considered significant. The results are based on three biological repeats performed in duplicates.

## 3. Results

### 3.1. Sub-Lethal Concentarations of Zn^2+^ Ions Inhibit Biofilm Formation by B. subtilis

**Figure 1:**
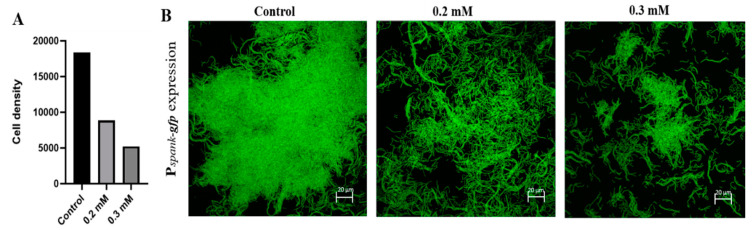
Inhibitory effect of Zn^2+^ ions on biofilm formation by *B. subtilis*. Zinc ions inhibited biofilm bundle formation by the bacterial cells grown in the presence of ZnCl_2_; Demonstrated by calculation of cells density (**A**) and shown by confocal laser scanning microscopy (CLSM) imaging of fluorescently tagged *B. subtilis* cells (**B**), following an overnight incubation in Lysogeny broth (LB) supplemented with 3% lactose at 23 °C (with 50 rpm shaking). Scale bar–20 μm.

This investigation was initiated to determine the effect of Zn^2+^ ions on formation of biofilm bundles during growth of bacterial cells in biofilm inducing conditions, over-night in the presence of ZnCl_2_ in concentrations of either 0.2 or 0.3 mM. Those levels of zinc were previously found in our lab to have no significance effect on bacterial growth (Figure S1). We visualized the effect of zinc microscopically by testing bundling phenotype of fluorescently tagged *B. subtilis* cells (YC161), which produce GFP constitutively. As seen in Figure 1, in the control sample, when bacteria cells were grown without zinc, they managed to form robust biofilm bundles. On the other hand, in the zinc treated samples, there was a notable inhibition of bundles formation, and the scarcely formed bundles were significantly smaller and not as dense as those found in the control sample.

### 3.2. Zn^2+^ Ions Inhibit Biofilm Formation through Downregulation of Genes Involved in Construction of Extracellular Matrix

**Figure 2:**
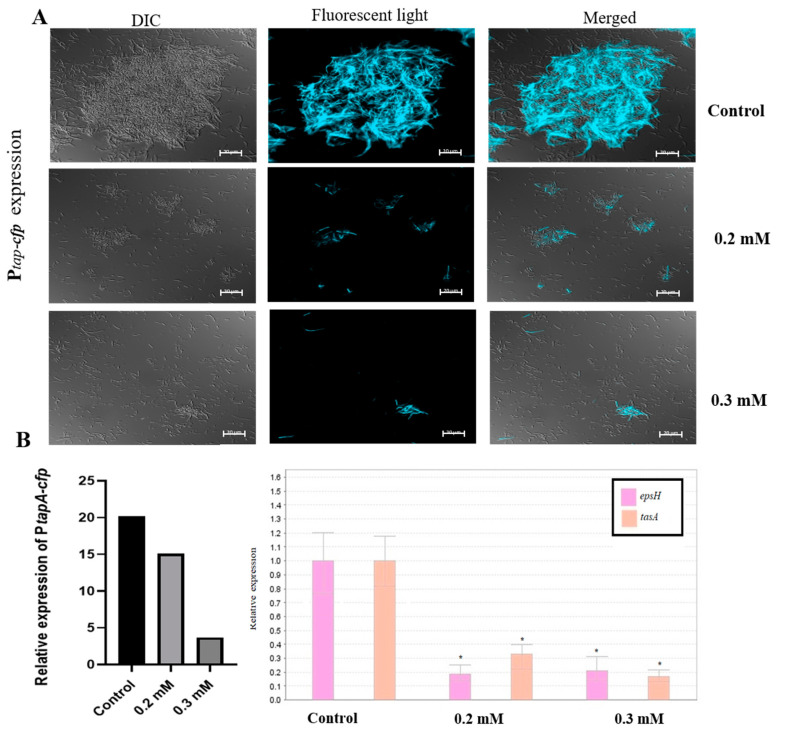
Zn^2+^ ions downregulate the expression of genes responsible for extracellular matrix production in *B. subtilis* biofilm. Shown by inverted florescence microscope images of *B. subtilis* cells bearing the P*tap-cfp* transcriptional fusion (**A**), measurement of cyan fluorescent protein (CFP) fluorescence intensity, and relative expression of *epsH* and *tasA* genes using real time RT-PCR analysis (**B**), following overnight incubation in LB supplemented with 3% lactose at 23 °C (with 50 rpm shaking), with or without the presence of ZnCl_2_. Scale bar—20 μm. * *p*-value < 0.05 for comparing with control.

We hypothesized that the dramatic decrease in biofilm formation observed in the presence of zinc could be a result of down-regulation of genes involved in matrix synthesis. To test this hypothesis, we first tested the effect of either 0.2 or 0.3 mM ZnCl_2_ on the expression of the *tapA-sipW-tasA* operon, which encodes the protein components of the extracellular matrix. For this purpose, we used the genetically modified *B. subtilis* strain (YC189), which express cyan-fluorescent protein (CFP) under the control of the *tapA* promoter. Therefore, the amount of the fluorescently visible cells represents the expression of the *tapA* promoter in the different samples. As shown in Figure 2A, expression of the *tapA* operon was notably reduced when bacteria cells were grown in the presence of zinc, comparing to the large fluorescent bundles received in the un-supplemented control sample. Bacterial cells in the treated samples seemed to be incapable of forming prominent bundles, and cells were mainly found spread out in a single cell mode. These results suggest that the anti-biofilm activity of zinc is through downregulation of the synthesis of extracellular matrix by *B. subtilis*. Next, in order to obtain quantitative information about the difference in *tapA* expression between the control and the supplemented samples, we performed real-time RT-PCR analysis. We intended to evaluate the expression of the *tasA* gene, while also examining the effect on the *epsH* gene, since both are genes of the matrix operons and in charge of producing the two main components of the biofilm extracellular matrix. As can be seen in Figure 2B, the expression of *epsH* and *tasA* genes was reduced in the presence of 0.2 and 0.3 mM ZnCl_2_. Measurement of CFP fluorescence intensity, indicating about the expression of the *TasA* gene, revealed a similar trend. These results support the data obtained at the microscopic visualization. Moreover, this experiment indicates that zinc not only down-regulates the expression of *tapA* operon, but also the expression of the *eps* operon.

### 3.3. Zn^2+^ Ions Disrupt Biofilm Bundles

**Figure 3:**
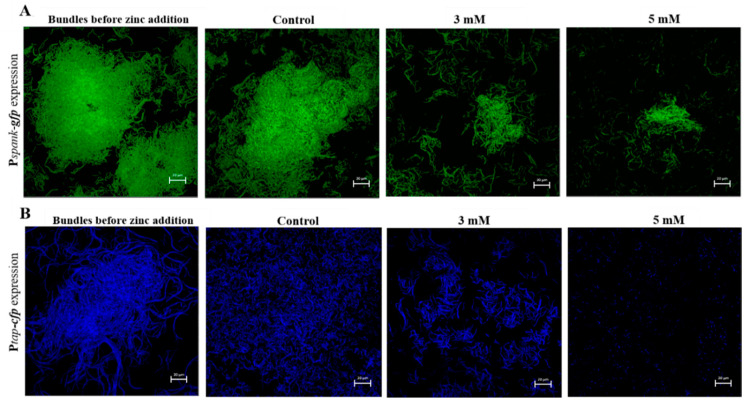
Antimicrobial effect of Zn^2+^ ions against *B. subtilis* biofilm bundles. Addition of zinc to the medium efficiently disrupted biofilm bundles formed overnight. Demonstrated in CLSM images of two fluorescently tagged *B. subtilis* strains: constitutively GFP producing (P*spank*-*gfp)* (**A**) and *tapA* promotor related CFP producing (P*tap*-*cfp*) (**B**) following 17 h incubation in LB supplemented with 3% lactose at 23 °C (with 50 rpm shaking), prior to zinc addition, and additional 5 h incubation in presence of ZnCl_2_. Scale bar—20 μm.

Since zinc addition has proven to be efficient in preventing biofilm formation, we next wanted to test whether Zn^2+^ ions are able to disrupt preformed biofilm. Biofilm-dispersion is generally a struggle for antimicrobials and disinfectant, mostly arising from their inability to fully penetrate the biofilm matrix once formed [8,9,10]. To investigate the effect of Zn^2+^ ions on disruption of biofilm, fluorescently tagged *B. subtilis* cells which produce GFP constitutively (YC161), were grown in biofilm promoting medium for 17 h. Next, we assured using microscopic visualization that bundles were in fact formed, and afterwards the cells were exposed to ZnCl_2_ at concentrations of 3 mM and 5 mM during incubation for 5 h. As shown in Figure 3A, bacteria cells were able to form large and layered biofilm bundles after the long overnight incubation, prior to zinc addition. Remarkably, as opposed to the prominent bundles found in the control, addition of zinc to the medium nearly completely abolished the previously present bundles.

We wanted to verify that the bacteria cells affected truly were cells in a biofilm state. For this reason, a similar experiment was conducted as described in the previous section, this time using bacteria cells of the fluorescently tagged (YC189) strain. As previously mentioned, this strain expresses CFP under the control of the *tapA* promoter, therefore when fluorescent bundles are visible it indicates that these bundles are in fact cells in biofilm mode, rather than a condensed group of vegetative bacterial cells. As shown in Figure 3B, fluorescently visible biofilm bundles were formed before the addition of zinc and continued to flourish in the zinc-absent (control) sample. On the other hand, once again, zinc addition efficiently reduced the size and density of the bundles. Notably, when bundles were exposed to zinc concentration of 5 mM, no prominent bundles were found, and it appears that this concentration completely disrupted any previously present biofilm bundles, leaving only parted and scattered bacteria cells. These results show that beside limiting bacterial growth and biofilm formation by *B. subtilis*, at certain concentrations zinc is also capable of affecting already formed biofilm bundles.

### 3.4. Morphological Changes in Bacterial Cells Exposed to Zinc

**Figure 4:**
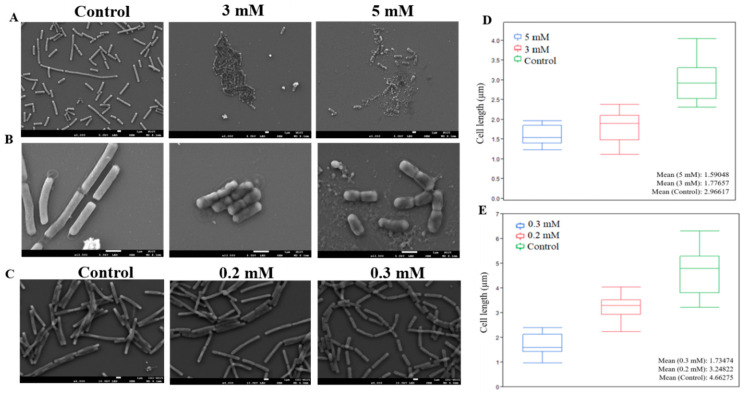
Zn^2+^ ions restrict elongation in *B. subtilis* cells. Scanning (SEM) images of *B. subtilis* cells following 4 h exposure to ZnCl_2_ in concentrations of 3 mM and 5 mM showing inhibition of cell elongation and abnormalities in cells shape. Overnight exposure to ZnCl_2_ in concentrations of 0.2 and 0.3 mM affected cell elongation. Images shown were taken at magnifications of ×3000 (**A**), ×13,000 (**B**) and ×4000 (**C**) with a Jeol JSM-IT-100 LV at 5.0–10.0 kV. Box plot graph displaying differences in cell size caused by exposure to 5 and 3 mM (**D**) or 0.2 and 0.3 mM ZnCl_2_ (**E**).

During this study, we tested two types of zinc levels upon bacterial cells—higher concentrations, aiming to kill bacteria or eliminate preformed biofilm bundles, and lower concentrations suitable for preventing biofilm formation. In order to gain a better understanding of zinc’s effect on bacterial cells, focusing on morphological changes, we sought to examine the effect of both concentrations using scanning electron microscopy (SEM). First, we tested the effect of higher levels of zinc, namely concentrations of either 3 or 5 mM ZnCl_2_. For this purpose, bacterial cells were grown for 4 h, with and without the presence of zinc, then taken for microscopic visualization and the imaged cells were measured for their length. As can be seen in Figure 4A,B, there were distinctive differences in cell shape and size between bacterial cells grown in the presence of zinc, comparing to those grown without. Cells in the control sample were much longer, growing to an average size of 2.96 µm. Cells grown without zinc also clearly matured into a healthy state and their cell wall seemed intact without any visible abnormalities. Contrarily, bacterial cells exposed to zinc were measured at much smaller size, reaching an average size of 1.77 and 1.59 µm, when treated with 3 and 5 mM, respectively (Figure 4D). Another visible difference between the zinc treated and untreated samples was achieved from a closer look at the cells (magnification of ×13,000). As shown in Figure 4B, there seems to be abnormalities in cell shape, both in the integrity of the cell wall and within the cell itself, which seemed to be deformed comparing to healthy cells in the control. Remarkably, when bacteria cells were exposed to 5 mM of ZnCl_2_, damage to cells was manifested with an evident loss of cell wall integrity and possible leakage of cellular content in some cells.

Similar morphological changes were observed in the presence of lower concentrations of Zn^2+^ ions. As can be seen in Figure 4C, bacterial cells grown overnight in the presence of 0.2 and 0.3 mM ZnCl_2_ were significantly shorter comparing to cells in the control. Since in this assay bacteria cells were grown overnight prior to microscopic visualization, untreated cells were longer than those in the previous experiment, reaching an average size 4.66 µm. On the other hand, growth was decreased in bacteria cells exposed to 0.2 mM of ZnCl_2_, and cells grew merely to the size of 3.24 µm on average. Growth was further restricted in the presence of 0.3 mM, and no cell was measured to reach the length of more than 2.5 µm, with average size of 1.73 µm (Figure 4E). Apart from changes in cell length, at these concentrations there were no visible differences in cell shape or any abnormalities comparing to the control, as opposed to the damage seen when we used higher levels of zinc. Additionally, no significant differences were observed regarding cell width, when bacteria were exposed to zinc in all tested concentrations. Therefore, it appears that zinc affect cell elongation in *B. subtilis* cells, however, it remains to be investigated whether Zn^2+^ ions affect division time. The different effect between the two levels of zinc seems to be their impact on cell wall integrity and deformation in cell shape and content, two phenotypes that were solely visible when bacteria cells were exposed to higher concentrations of zinc.

### 3.5. Bacterial Cells Exposed to Zinc are More Sensitive to Heat Treatment

**Figure 5:**
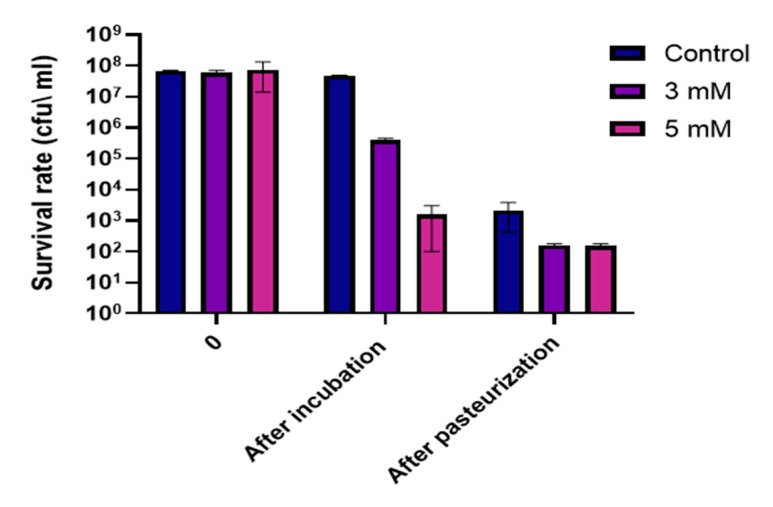
Exposure to Zn^2+^ ions decreases resistance of *B. cereus* cells to heat treatment. *B. cereus* cells incubated with 3 mM and 5 mM ZnCl_2_ were subjected to heat treatment performed at 72 °C for 30 s. Survival rate was determined using the CFU method. * *p*-value <0.05 comparing with control. Error bars represent standard deviation (SD).

In the light of finding that Zn^2+^ ions can undermine the ability to form and maintain stable biofilm structures by *B. subtilis*, we wanted to test whether incubation of bacteria with zinc prior to heat treatment will affect their ability to survive this type of stress. This experiment was conduct on two strains that are part of the *Bacillus* species: *Bacillus subtilis* NCIB3610 and *Bacillus cereus* ATCC 10987. As previously mentioned, members of the *B. cereus* and *B. subtilis* group are the most important spoilage microorganisms in the dairy environment [17]. To test the effect of Zn^2+^ ions on the survival of *B. cereus*, bacteria was grown for 5 h, then incubated with various concentrations of zinc for 3 h. Bacterial levels were measured before and after incubation with zinc, and survival rates were measured following the heat treatment. As can be seen in Figure 5, there was a significant concentration dependent reduction of 2-log and 5-log in bacterial level prior to pasteurization. Post pasteurization sampling showed a 1-log reduction in survival rate of *B. cereus* cells exposed to both 3 and 5 mM ZnCl_2_, comparing to bacterial level in the control sample. These results can be an example for one way in which zinc addition can be used in an applicative way in the food processing industry, specifically in the dairy industry. Furthermore, in the case of 5 mM ZnCl_2_, bacterial levels dropped down significantly after a 3 h incubation even without heat treatment. Overall, Zn^2+^ ions showed a strong antimicrobial effect against bacterial cells, even before the application of heat treatment. This result opens opportunity for developing novel antimicrobial technologies as well as a possible energy-saving antimicrobial alternative for the dairy industry.

## 4. Discussion

This study was designed to test whether zinc as an antimicrobial can provide a solution to microbial contaminations that frequently occur in the dairy industry, specifically regarding contaminations emerging from bacterial presence of members of the *Bacillus* spp [14,39]. Since biofilms are a great concern in various food sectors, many studies have been done in order to gain a better understanding of their development, subsequently coming up with fitting countermeasures [3]. In regard to the dairy industry, it appears that a main source for contamination of dairy products is derived from biofilms populating all types of product contact surfaces at various stages of milk production and processing [3,12,40]. A big issue in the food industry when dealing with cleaning procedures comes from the notion that conventional cleaning and disinfection regimes can be ineffective in controlling biofilm formation due to failure of the antimicrobial agents to fully penetrate the biofilm matrix [8,9,10]. Therefore, a sensible solution could be incorporation of a substance that damages the exopolysaccharides matrix as part of the cleaning processes, hence leaving the cells within the biofilm vulnerable to mechanic and chemical stresses induced by the cleaning procedure. In our study, we found that zinc can inhibit biofilm formation and disrupt *B. subtilis* biofilm bundles, seemingly by down regulation in the synthesis of extracellular matrix components. These findings can mark zinc as a substance that could provide a solution to this widespread food industry concern, specifically suitable for the dairy industry.

The mechanism behind the antibiofilm activity of the Zn^2+^ ions has yet to be fully characterized, but it has been suggested that zinc could interact with components in the matrix [31]. This assumption has proved to be right in the case of several species, concerning their strain-specific matrix composition. Research on the effect of zinc on *B. subtilis* biofilm is lacking and focused mainly around the use of ZnO-nanoparticles. One study found that ZnO-nanoparticles prevented *B. subtilis* biofilm formation by reducing EPS production [41]. This finding correlates with our results, since a decrease in the expression of the *epsH* gene was also achieved after incubation with ZnCl_2_. An extension to this knowledge is provided by our study, since we also observed a decline in the expression of the *tasA* gene. TasA forms a network in the biofilm matrix, critical for structural integrity as well as for the development of biofilm architecture. Combined with the reduction in *epsH* expression, it seems that zinc’s anti-biofilm mode of action involves interfering with the construction of an extracellular matrix. A recent study demonstrated the effect of Zn^2+^ ions on biofilm formation in *B. amyloliquefaciens*, a root-colonizing bacterium [42]. Similar to our results, the presence of zinc inhibited biofilm formation by interfering with the synthesis of the same extracellular matrix components. Furthermore, the study also showed that Zn^2+^ ions indirectly inhibited Spo0F, a response regulator of the matrix operons, by inhibiting Mn^2+^ uptake, necessary for its activity. Likewise, Mn^2+^ is known to be essential for proper biofilm development in *B. subtilis* [43]. This suggests that interference with Mn^2+^ homeostasis, as a response to excessive Zn^2+^ uptake, might have been another possible antibiofilm pathway for zinc.

Investigation of the molecular basis of zinc’s effect on *B. subtilis* cells was another aim set for this study. It is difficult to infer about the effect of Zn^2+^ ions on bacterial cells from studies conducted using ZnO, especially in the form of Zn-nanoparticles. ZnO antimicrobial mechanism partly relies on the generation of hydrogen peroxide (H2O2) [44]. Given the different chemical composition, this cannot be the case for bacterial cells treated with ZnCl_2_, as done in our study. Several previous studies suggested that the mechanism of nanoparticles toxicity may be related to production of reactive oxygen species (ROS), which can indirectly damage cell membranes, possibly through lipid peroxidation. For example, damage and disorganization in the cell wall were observed in bacteria exposed to MgO and ZnO NPs [45,46]. In a study conducted using Ag-ZnO nanocomposite against *S. aureus* and *E. coli* cells, upon treatment bacteria demonstrated strong evidence of membrane disorganization and increased roughness which have led to leak-out of the intracellular components, causing shrinkage of cell and finally cell lysis in both strains [47]. In our study, *B. subtilis* cells grown in the presence of ZnCl_2_ showed a phenotype of possible cell wall damage, a possible leakage of intracellular components and cell lysis when cells were treated with 5 mM ZnCl_2_. The formerly mentioned study suggested that the role of Zn^2+^ ions in inducing cell death could be either strong electrostatic interaction between the ions and the negatively charged cell membrane of the bacterial cells or via production of intracellular ROS, based on previously reported literature [48,49]. Furthermore, it was demonstrated that ZnO is more effective in the killing of Gram-positive bacteria rather than Gram-negative, seemingly due to their simpler cell membrane structure. Hence, suggesting that the antimicrobial effect of zinc on the bacterial cell is strongly related to direct and indirect influence on membrane integrity. Disturbance of metal homeostasis and the subsequent damage to cell membrane appears to be a central notion in the search for zinc’s antimicrobial mechanism [50]. This concept was demonstrated in *B. subtilis* cells that exhibited heme toxicity after being treated with excessive levels of Zn^2+^ ions, seemingly due to mismetallation of Fe^2+^ [51]. Heme toxicity induces damage that is primarily localized to the cell membrane, as shown in *S. aureus* [52]. *B. subtilis* cells exposed to zinc were also significantly shorter than those grown without zinc. It is unclear whether the noted decrease in cell elongation derives from possible damage to cell membrane or whether it results from other targets that can be affected by the presence of zinc.

Finally, we wanted to examine the potential of zinc-based antibacterial treatment, in the context of milk and its derivatives. Vegetative cells of *B. cereus* and *B. subtilis* were found to be more sensitive to heat treatment after an incubation with zinc prior to pasteurization. In the dairy industry, pasteurization process is the widely adopted technology for reducing microbial load, taken in order to make milk safe for consumption and to extend the shelf life of dairy products. In this research, we found a 1-log reduction in the survival rate of *Bacillus* cells past heat treatment, when exposed to 3 and 5 mM ZnCl_2_. Zinc’s antimicrobial effect is thought to be the result of multiple targets and cellular interactions. Therefore, we cannot fully conclude why the presence of zinc made bacteria more susceptible to heat treatment. However, based on our observations, zinc was not found to significantly affect sporulation in either *B. subtilis* or *B. cereus*. Therefore, it is believed that the decrease in cell counts post pasteurization has been derived from zinc’s effect against vegetative cells. Regardless, we further speculate that increased sensitivity of bacteria to heat treatment in the presence of Zn^2+^ ions can possibly lead to improvement of antibacterial industrial procedures, and therefore enhancement of microbial safety and quality of dairy products and perhaps additional foods. One possible future target can be beverages, such as fruit and vegetable juices, also known to struggle with issues of contaminations and spoilage.

During the last decades, the food market has undergone considerable transformations in order to satisfy the requirements of consumers looking for more ‘natural’ products that are minimally processed with no/fewer synthetic additives [53,54]. In order to meet these demands, manufacturers are trying to find a balance between ensuring food safety without compromising products’ nutritional quality. Consequently, several techniques can be used for minimally processed foods: non-thermal treatments, low-temperature storage, new packaging, and treatment with natural antimicrobials [53,54]. Since zinc supplementation in concentration of 5 mM has led to a notable reduction in *B. cereus* and *B. subtilis* levels before pasteurization, it is possible that zinc can act as a non-thermal way of treatment, while also being a natural antimicrobial. Regarding the use of zinc as a natural antimicrobial substance, further investigation is required in order to assert whether addition of zinc is sufficiently effective, basing on comparison with the currently used synthetic preservatives. Overall, it appears that incorporation of zinc into the final product can strengthen the microbial safety of the product and at the same time provide desirable amounts of this essential mineral when consumed.

Zinc is an essential trace element not only for humans, but for all organisms. It is indispensable for basic human health, since it is a component of more than 300 enzymes and an even greater number of other proteins [55]. Compared to other metal ions with similar chemical features, zinc is relatively harmless. This is a result of zinc homeostasis, which allows for the efficient handling of any excess of orally ingested zinc [56]. According to the Toxnet database of the U.S. National Library of Medicine, the oral LD_50_ for zinc is close to 3 g/kg body weight. Long-term, high-dose zinc supplementation has been found to interfere with the uptake of copper. Hence, zinc intoxication is possible; however, it is considered to be a rare event and can only result from exposure to high doses. Whereas intoxication by excessive exposure is rare, zinc deficiency is widespread and can impact on growth, neuronal development, and immunity among other [57]. Overall, it seems that zinc deficiency is a far more common risk to human health than intoxication. However, when food supplementation is the target, dietary recommendations and daily intake limitations should definitely be taken into account when choosing the appropriate zinc levels in the final product.

## Supplementary Materials

The following are available online at https://www.mdpi.com/2304-8158/9/8/1094/s1, Figure S1: growth curve of *B. subtilis* NCIB3610 in the presence of ZnCl_2_ in concentrations of 0.2 and 0.3 mM. Table S1: Primers used for RT-PCR analyses.
