# Supplementary material for: Mitigating Milk-Associated Bacteria through Inducing Zinc Ions Antibiofilm Activity
Source: Foods. 2020 Aug 11;9(8):1094. doi: 10.3390/foods9081094 (PMC7466369; doi:10.3390/foods9081094)
Supplement: Supplementary file 1 [file foods-09-01094-s001.pdf]

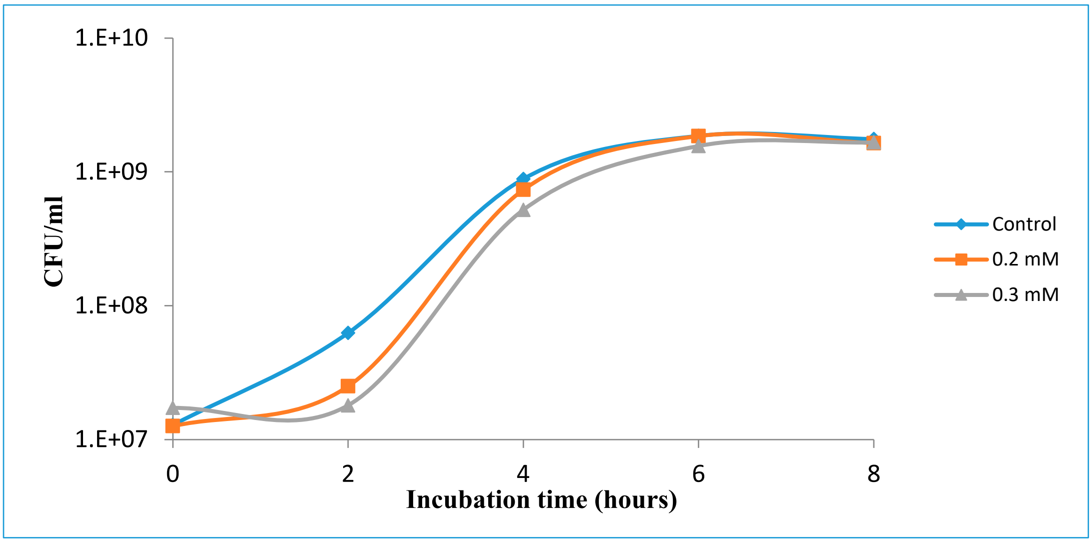

Figure 1. Effect of low concentrations of zinc on bacterial growth. Growth curve analysis was done on *B. subtilis* cells incubated in LB for 8 h at 37°C with 150 rpm, with or without the presence of $ZnCl_2$. Viable cell count was done using CFU methods.

Table 1. Primers used for RT-PCR analyses.

Gene	sequence (5′-3′)
rpoB-F	5′ TGCCGGTTACGGTTCTTTTG 3′
rpoB-R	5′ TGTCGCTGTTTTCTGTGTTATCTTTAT 3′
16S rRNA-F	5′ GCGAAGTGCGGGTGATTT3 3′
16S rRNA-R	5′ GCAGTCTATGTGTTACCGTTACCT 3′
tasA-F	5′ CCGCTCCTGAATATGATGGT 3′
tasA-R	5′ GCCGTTCCACTGTGTAGCTT 3′
epsH-F	5, ACTCTGACATTGCCCAAACC 3′
epsH-R	5′ GCCCTGAAGCTGAAAAACTG 3′
